# Long Non-coding RNA RMRP in the Pathogenesis of Human Disorders

**DOI:** 10.3389/fcell.2021.676588

**Published:** 2021-04-30

**Authors:** Bashdar Mahmud Hussen, Tahereh Azimi, Hazha Jamal Hidayat, Mohammad Taheri, Soudeh Ghafouri-Fard

**Affiliations:** ^1^Department of Pharmacognosy, College of Pharmacy, Hawler Medical University, Erbil, Iraq; ^2^Department of Medical Genetics, Shahid Beheshti University of Medical Sciences, Tehran, Iran; ^3^Department of Biology, College of Education, Salahadddin University-Erbil, Erbil, Iraq; ^4^Urology and Nephrology Research Center, Shahid Beheshti University of Medical Sciences, Tehran, Iran

**Keywords:** long non-coding RNA RMRP, cancer, expression, biomarkers, cell lines

## Abstract

RNA component of mitochondrial RNA processing endoribonuclease (RMRP) is a non-coding transcript firstly acknowledged for its association with the cartilage-hair hypoplasia (CHH) syndrome, a rare autosomal recessive condition. This transcript has been spotted in both nucleus and mitochondria. In addition to its role in the pathogenesis of CHH, RMRP participates in the pathogenesis of cancers. Independent studies in bladder cancer, colon cancer, hepatocellular carcinoma, lung cancer, breast carcinoma and multiple myeloma have confirmed the oncogenic effects of RMRP. Mechanistically, RMRP serves as a sponge for some miRNAs such as miR-206, miR-613, and miR-217. In addition to these miRNAs, expressions of tens of miRNAs have been altered following RMRP silencing, implying the vast extent of RMRP/miRNA network. In the present narrative review, we explain the role of RMRP in the development of cancers and some other non-malignant disorders.

## Introduction

Although protein-coding genes comprise a minor portion of the mammalian genome, it has been revealed that the vast majority of these genomes is transcribed at some level (Carninci et al., 2005; Birney et al., 2007). It has also been speculated that considerable portion of these transcripts are likely functional (Mattick et al., 2010). Notably, the ENCODE consortium has described assignment of “biochemical functions” to approximately 80% of the genome (Consortium, 2012). This group of non-coding RNAs (ncRNAs) includes several members ranging from the lately described abundant ribosomal RNAs (rRNAs) (Stark et al., 1978), small nuclear RNAs (snRNAs) and small nucleolar RNAs (snoRNAs) to those with recently appreciated regulatory RNAs, namely microRNAs (miRNAs) and long non-coding RNAs (lncRNAs) (Palazzo and Lee, 2015). miRNAs have sizes about 22 nucleotides and regulate expression of genes mostly through binding with 3′ untranslated regions of target transcripts (Macfarlane and Murphy, 2010). LncRNAs have sizes over 200 nucleotides and are transcribed by RNA Polymerase (RNA Pol) II and RNA Pol III. They modulate numerous cellular processes including histone modification, DNA methylation, and transcription of genes through modulating chromatin configuration and DNA accessibility (Dahariya et al., 2019).

RNA component of mitochondrial RNA processing endoribonuclease (RMRP) is a transcript with wide expression in diverse tissues obtained from human and mice species (Rosenbluh et al., 2011). RMRP has a regulatory role in the processing of RNA in both mitochondrial and ribosomal compartments (Hermanns et al., 2005). RMRP transcripts have been detected in both mitochondria and nucleus (Chang and Clayton, 1987; Rosenbluh et al., 2011). This lncRNA has a remarkable role in the primary stages of mice development (Rosenbluh et al., 2011). In humans, mutations in the *RMRP* gene has been associated with cartilage-hair hypoplasia (CHH) syndrome, a multi-systemic disorder that is inherited via an autosomal recessive mode. Clinical characteristics of CHH are unbalanced short stature, fine and scant hair, defects in cellular immunity and susceptibility to cancer which are related to defects in expression of RMRP (Hermanns et al., 2005). Although immunodeficiency is a possible underlying mechanism of malignancy in these patients, many CHH cases with no history of immunodeficiency has developed neoplastic conditions such as lymphoma, implying a multifactorial basis for development of malignancy in CHH (Vakkilainen et al., 2019b). Epstein-Barr virus infection can explain some cases of lymphoproliferative diseases in CHH (Taskinen et al., 2013; Sathishkumar et al., 2018). Yet, not all lymphoproliferative disorders are related with this virus (Nguyen et al., 1718). In addition to defects in response to viral infections, chromosomal instability (Hauck et al., 2018) and impaired telomere function (Kostjukovits et al., 2017) might explain the increased risk of malignancy in CHH. A high throughput expression assay in CHH fibroblasts has shown abnormal expression of tens of gene. Notably, under-expressed genes have been functionally associated with cell cycle. Moreover, regulatory pathways of apoptosis, bone and cartilage development, and functions of lymphocyte, and PI3K-Akt cascade have been among other dysregulated mechanisms. CHH cells have exhibited delays in the transition from G2 stage to mitosis (Vakkilainen et al., 2019a). Experiment in Zebrafish model of CHH produced by *Rmrp* knock-down has verified the role of over-activation of Wnt/β-Catenin signaling in disruption of chondrogenesis and bone ossification (Sun et al., 2019). Although RMRP has been shown to bind with the mitochondrial RNA processing complex RNase MRP (Chang and Clayton, 1987), there is no obvious mitochondrial deficiencies in CHH patients. In conjunction with hTERT, RMRP can make an RNA dependent RNA polymerase which transforms single stranded RMRP transcript into double stranded form (Maida et al., 2009). Over-expression of RMRP has been demonstrated in a wide spectrum of human malignancies (Tang et al., 2019). In the present narrative review, we explain the role of RMRP in the development of cancers and some other non-malignant disorders.

## Malignant Conditions

### Cell Line Studies

**FIGURE 1:**
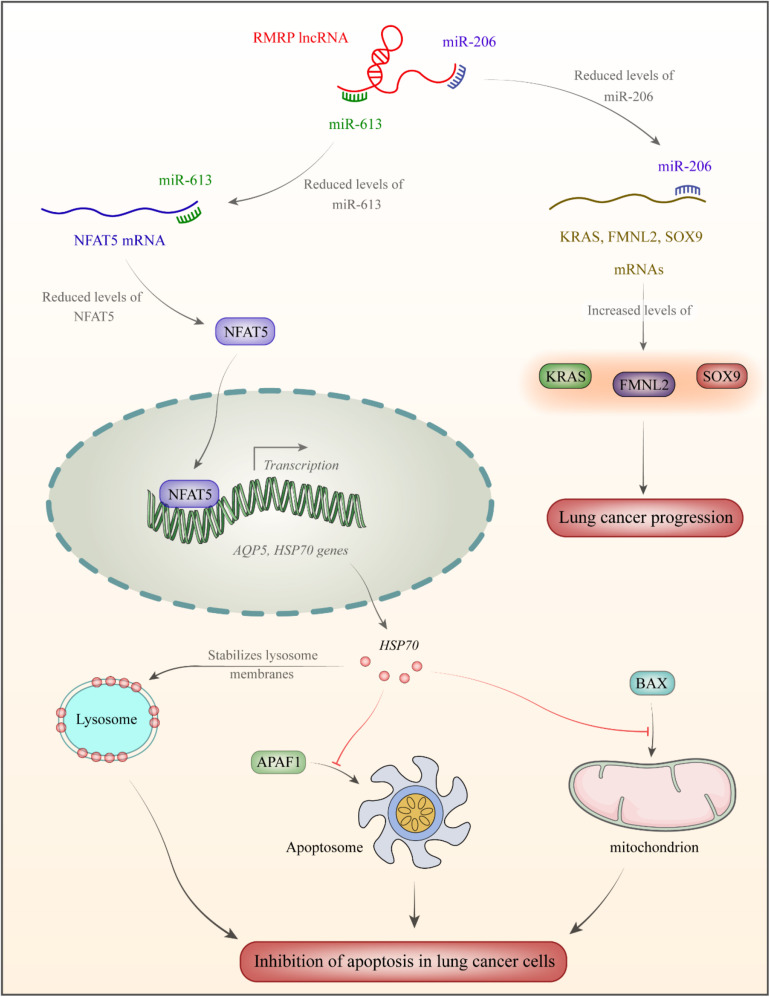
RMRP has been over-expressed in lung cancer (Shao et al., 2016). This lncRNA acts as a sponge for miR-613 and miR-206. miR-206 binds with 3′UTR of NFAT5 transcription factor (Yang et al., 2020). This transcription factor increases expression of AQP5 (Guo and Jin, 2015) and the heat shock protein HSP70 which increases stability of lysosome membrane (Boya and Kroemer, 2008). As a member of HSP family, it is produced following exposure to cellular stressful situations such as excessive heat/cold, ultraviolet light and in the course of wound healing or tissue remodeling. HSP70 have chaperone functions through stabilizing proteins to certify correct folding or assisting in protein refolding (Rosenzweig et al., 2019). Moreover, HSP70 inhibits APAF1 binding with apoptosome and suppresses BAX binding to mitochondria, thus decreasing apoptosis rate in lung cancer cells (Wu et al., 2017). miR-206 binds with 3′UTR of SOX9, FMNL2, and KRAS. Down-regulation of miR-206 enhances protein levels of these genes in lung cancer (Shao et al., 2016).

In the cholangiocarcinoma cells, up-regulation of RMRP is associated with down-regulation of miR-217, a miRNA that is sequestered by RMRP (Tang et al., 2019). RMRP silencing has resulted in up-regulation of several miRNAs such as hsa-miR-33a-3p, hsa-miR-186-5p, and hsa-miR-216a-5p, while down-regulation of hsa-miR-345-5p, hsa-miR-1275, and hsa-miR-4454 (Tang et al., 2019). RMRP silencing can suppress proliferation of cholangiocarcinoma cells, stimulate apoptosis in these cells, and block them in the G0/G1 stage (Tang et al., 2019). In lung cancer cells, RMRP silencing evidently reserved cell proliferation, migration, and invasiveness, while blocking cell cycle transition. miR-1-3p has been identified as a target of RMRP in these cells (Tang et al., 2019). Figure 1 displays the mechanism of RMRP-mediated oncogenesis in lung cancer.

In the bladder cancer cell lines, RMRP can enhance proliferation, migration potential and invasiveness of cells through modulating expression of miR-206 (Cao et al., 2019), a tumor suppressor miRNA that induces cell cycle arrest (Huang et al., 2016). Based on the presence of the β-catenin/TCF and YAP/TBX5 constituents in the upper parts of the *RMRP* gene, expression of RMRP might be associated with the cancer-associated pathways, Wnt/β-catenin and Hippo/YAP. Functional studies have shown that induction of Wnt signaling enhances expression of RMRP via β-catenin and YAP nuclear factors (Park and Jeong, 2015). In hepatocellular carcinoma cells, RMRP silencing has precluded cell proliferation, migration and invasive properties, while stimulating cell cycle arrest. These effects are mediated by sponging miR-613 (Zhou et al., 2019). Another study in this type of cancer has shown the role of RMRP in sequestration of miR-206 and activation of TACR1/Erk1/2 pathway (Hongfeng et al., 2020). However, Shao et al. (2020) have demonstrated down-regulation of RMRP in the hepatocellular carcinoma cells. Enforced over-expression of RMRP in these cells enhanced apoptosis rate of these cells through modulating miR-766 expression (Shao et al., 2020). RMRP silencing in gastric cancer cells suppresses cell proliferation via modulation of miR-206 and subsequent regulation of cell cycle transition through modulation of Cyclin D2 (Shao et al., 2016). In the neuroblastoma cells, the oncogenic effects of RMRP are mediated through sequestering miR-206 and enhancing expression of TACR1 (Pan et al., 2019). In the papillary thyroid carcinoma cells, expression of RMRP has been increased while expression of miR-675 has been diminished. MAPK1 has been identified as a target of miR-675 in these cells (Wang et al., 2019). A brief review of investigations that appraised RMRP expression in cancer-derived cell lines is presented in Table 1.

**TABLE 1:** Brief results of studies which gauged expression of RMRP in cancerous cell lines (Δ: knock-down).

**Cancer types**	**Targets/regulators and signaling pathways**	**Assessed cell lines**	**Function**	**References**
Bladder cancer	miR-206	BIU-87, T24, and SV-HUC-1	Δ RMRP: ↓ cell proliferation, migration, and invasion	Cao et al. (2019)
Colorectal cancer	Wnt and Hippo signaling pathways (β-catenin/YAP/TBX5)	SW480, HT-29, HCT116, HEK293, HEK293T, and Beas-2B	–	Park and Jeong (2015)
Hepatocellular carcinoma	miR-613	Hep3B, HCCLM3, and HL-7702	Δ RMRP: ↓ cell proliferation, migration, and invasion	Zhou et al. (2019)
	miR-206/TACR1	SMMC-7721, Bel-7402, MHCC-97, HepG2, Hep3B, Huh-7, and HL- 7702	Δ RMRP: ↓ cell proliferation, migration, invasion, and ↑ apoptosis	Hongfeng et al. (2020)
	miR-766	Hep3B, HepG2, MHCC97H, HuH7, and HL-7702	↑ RMRP: ↓ cell proliferation, migration, invasion, and ↑ apoptosis	Shao et al. (2020)
Cholangiocarcinoma	miR-217	HCCC-9810 and RBE	Δ RMRP: ↓ cell proliferation, migration, invasion and ↑ apoptosis	Tang et al. (2019)
Gastric cancer	miR-206/Cyclin D2	AGS, BGC-823, HGC-27, MGC-803, SGC-7901, and GES-1	Δ RMRP: ↓ cell proliferation and ↑ apoptosis	Shao et al. (2016)
Glioma	–	U87 and U251	Δ RMRP: ↓ cell proliferation, migration, invasion, and ↑ apoptosis	Feng et al. (2017)
Neuroblastoma	miR-206/TACR1 and ERK1/2 pathway	NB-1, SK-N-AS, and HEK293T	Δ RMRP: ↓ cell proliferation, migration, and invasion	Pan et al. (2019)
Non-small cell lung cancer	miR-206/KRAS, FMNL2 and SOX9	A549, SPC-A1, H1299, H23, and 16HBE	↑ RMRP: ↑ cell proliferation, colony formation, and invasion	Shao et al. (2016)
	miR-613/NFAT5	HCC827, 16HBE, H1299, H1975, A549, and HCT116	Δ RMRP: ↓ cell proliferation, migration, and invasion	Yang et al. (2020)
	miR-1-3p/ANXA2	A549, Calu1, H1299, H460, and BEAS-2B	Δ RMRP: ↓ cells proliferation, migration and invasion	Tang et al. (2019)
Papillary thyroid cancer	miR-675/MAPK1	HTH83, BCPAP, and TPC-1 and HT-ori3	Δ RMRP: ↓ cell proliferation, migration and invasion	Wang et al. (2019)
Multiple myeloma	miR-34a-5p/c-Myc	NCI-H929, RPMI-8226 and NPCs	Δ RMRP: ↓ cell proliferation and ↑ apoptosis	Kong et al. (2019)

### Animal Studies

*In vivo* assays have verified the cancer-promoting effects of RMRP. RMRP silencing has attenuated tumorigenesis process in xenograft model of liver cancer through modulation of miR-613 expression (Zhou et al., 2019). However, another *in vivo* study demonstrated the opposite role for RMRP in the pathogenesis of hepatocellular carcinoma through modulation of miR-766 (Shao et al., 2020). RMRP silencing has decreased the rate of growth of cholangiocarcinoma in animal models in association with the frequency of Ki-67-positivite cells in these tumors (Tang et al., 2019). In the xenograft model of gastric cancer, RMRP silencing attenuated tumor growth via modulation of miR-206 expression (Shao et al., 2016). A brief record of *in vivo* studies is shown in Table 2.

**TABLE 2:** Brief reports of studies which assessed function of RMRP in cancer animal models (Δ: knock down or deletion).

**Cancer types**	**Animal models**	**Function**	**References**
Hepatocellular carcinoma	Male athymic Balb/c nude mice	Δ RMRP: ↓ tumor size and weight	Zhou et al. (2019)
	Male Balb/c nude mice	↑ RMRP: ↓ tumor volume and weight	Shao et al. (2020)
Cholangiocarcinoma	Male nude mice	Δ RMRP: ↓ tumor volume and weight	Tang et al. (2019)
Gastric cancer	Male BALB/c nude mice	Δ RMRP: ↓ tumor growth	Shao et al. (2016)
Neuroblastoma	Female athymic nude mice	Δ RMRP: ↓tumor volume and weight	Pan et al. (2019)
Non-small cell lung cancer	Male Balb/c nude mice	Δ RMRP: ↓ tumor growth, migration and invasion	Yang et al. (2020)
Multiple myeloma	Male BALB/c-nude mice	Δ RMRP: ↓ tumor growth	Kong et al. (2019)

### Clinical Studies

Cao et al. (2019) have confirmed up-regulation of RMRP in bladder cancer samples in comparison with the nearby non-cancerous samples. Expression levels of RMRP have been correlated with tumor dimensions, lymph node metastasis and outcome of cancer in these patients (Cao et al., 2019). Levels of RMRP have also been up-regulated in clinical samples of colorectal and breast cancer patients (Park and Jeong, 2015). Over-expression of RMRP has also been reported in patients with hepatocellular carcinoma in correlation with tumor aggressiveness and adverse clinical outcome (Zhou et al., 2019). Accordingly, over-expression of RMRP has been identified as an indicator of poor prognosis in these patients (Hongfeng et al., 2020). In contrast with these studies, Shao et al. (2020) have reported down-regulation of RMRP in clinical samples obtained from patients with hepatocellular carcinoma. Under-expression of RMRP in these patients has been reported to be associated with low survival (Shao et al., 2020). RMRP expression has been increased in non-small cell lung cancer tissues in association with high clinical stage and poor patients’ outcome (Tang et al., 2019). Expression of RMRP has been elevated in tissue, plasma and gastric juices obtained from patients with gastric cancer in association with Borrmann type and metastatic capacity. Notably, they reported suitability of levels of RMRP in plasma and gastric juice for diagnosis of gastric cancer (Shao et al., 2016). In the glioblastoma samples, RMRP over-expression has been associated with high tumor grade, low Karnofsky Performance Score and poor clinical outcome (Feng et al., 2017). A summary of experiments which appraised transcript levels of RMRP in clinical specimens from cancer patients is shown in Table 3.

**TABLE 3:** Outlines of studies reported expression of RMRP in cancerous clinical samples (OS: overall survival, ANTs: adjacent normal tissues).

**Cancer types**	**Samples**	**Expression (tumor vs. normal)**	**Kaplan-Meier analysis**	**References**
Bladder cancer	91 paired of BC tissues and adjacent tissues	Up	Lower RMRP expression correlated with better prognosis.	Cao et al. (2019)
Colorectal cancer (CRC)	8 paired of CRC tissues and ANTs	Up	–	Park and Jeong (2015)
Hepatocellular carcinoma (HCC)	52 paired of HCC tissues and ANTs	Up	Higher RMRP expression levels correlated with shorter OS in HCC patients compared with lower group.	Zhou et al. (2019)
	42 paired of HCC tissues and matched normal tissues	Up	RMRP high expression levels associated with decrease of OS rate in HCC patients.	Hongfeng et al. (2020)
	40 paired of HCC tissues and ANTs	Down	High RMRP expression levels correlated with better 5-years and median survival rates in HCC patients.	Shao et al. (2020)
Cholangiocarcinoma (CCA)	33 paired of CCA tissues and ANTs	Up	Patients with lower RMRP expression levels had longer OS.	Tang et al. (2019)
Gastric cancer (GC)	132 paired of GC and non-tumorous tissues	Up	–	Shao et al. (2016)
Glioma	39 glioma tissues and 11 normal brain tissues	Up	Higher RMRP expression levels correlated with poorer OS in glioma patients than that of the lower group.	Feng et al. (2017)
Neuroblastoma	44 paired of neonatal neuroblastoma tissues and ANTs	Up	Higher RMRP expression levels associated with poor prognosis in neonatal neuroblastoma patients.	Pan et al. (2019)
Non-small cell lung cancer (NSCLC)	Plasma specimens from 63 NSCLC patients and 33 cancer-free smoker individuals	Up	–	Lin et al. (2018)
	35 paired of lung adenocarcinoma tissues and ANTs	Up	–	Shao et al. (2016)
	80 paired of NSCLC tissues and ANTs	Up	RMRP high expression levels associated with decrease of OS rate in NSCLC patients.	Yang et al. (2020)
	38 paired of NSCLC tissues and ANTs	Up	Higher RMRP expression levels correlated with poorer OS in NSCLC patients compared to lower group.	Tang et al. (2019)
Breast cancer (BC)	8 paired of BC tissues and ANTs	Up	–	Park and Jeong (2015)
Papillary thyroid cancer (PTC)	57 paired of PTC tissues and ANTs	Up	–	Wang et al. (2019)
Multiple myeloma (MM)	Bone marrow specimens from 28 MM patients and 10 healthy donors	Up	High RMRP expression levels correlated with shorter disease-free survival and OS in MM patients.	Kong et al. (2019)

Somatic mutations in the promoter region of this lncRNA have been firstly demonstrated in breast cancer (Rheinbay et al., 2017). The hotspot mutation region in this study has been further assessed in another cohort of patients with diverse types of cancers showing the presence of RMRP promoter mutations in two gastric cancer samples, a colon carcinoma sample and a sarcoma. Notably, none of these mutations has been formerly reported in breast cancers (Son et al., 2020). Table 4 shows the diverse mutations identified in the promoter region of RMRP in different solid and hematologic tumors.

**TABLE 4:** Summary of studies that have analyzed RMRP promoter mutations in solid and hematologic tumors.

**Type of cancers**	**Number of tumors**	**Wild type**	**Mutation**	**Location**	**Mutation (%)**	**References**
Gastric carcinoma	230	228	2	Chr9:35,658,037dupA Chr9: 35,658,174dupT	0.9	Son et al. (2020)
Colorectal carcinoma	388	387	1	Chr9:35,658,167G > T	0.3	Son et al. (2020)
Sarcoma	70	69	1 (malignant fibrous histiocytoma)	Chr9.35,658,015_35,658,031	1.4	Son et al. (2020)
				dupCACGTCCTCAGCTTCAC (17 bp)		
Breast cancer	360	–	–	Chr9:35658033 G > A Chr9:35658043 T > G	–	Rheinbay et al. (2017)
Adulthood AML	200	199	1 (AML with multilineage dysplasia)	g.35,658,020_35,658,039 dup CCTCAGCTTCACAGAGTAGT (20 bp)	0.5	Son et al. (2019)
Adulthood ALL	150	149	1 (B-ALL)	g.35,658,017_35,658,037 dup CGTCCTCAGCTTCACAGAGTA (21 bp)	0.7	Son et al. (2019)
Childhood ALL	200	199	1 (B-ALL)	g.35,658,029_35,658,041	0.5	Son et al. (2019)
				dupCACAGAGTAGTAT (13 bp)		
Multiple myeloma	75	74	1	g.35,658,015_35,658,031 dup CACGTCCTCAGCTTCAC (17 bp)	0	Son et al. (2019)

### Non-malignant Disorders

Han et al. (2020) have assessed the role of RMRP in lipopolysaccharide (LPS)-associated sepsis. They reported down-regulation of RMRP following LPS exposure. This down-regulation has been accompanied by significant reductions in MMP and mitochondrial cytochrome C levels, increased cardiomyocyte apoptosis, over-production of reactive oxygen species, up-regulation of cytochrome C in the cytoplasmic compartment, and over-production of caspase-3 and caspase-9 and NF-κB p65 subunit. Their *in vivo* experiments also verified the role of RMRP in the suppression of LPS-associated apoptosis and mitochondrial defects through sponging of miR-1-5p (Han et al., 2020). NF-κB p65 is a subunit of NF-κB transcription complexes (Sun, 2011). In fact, NF-κB family includes several transcription factors regulating expression of genes that partake in numerous crucial physiological responses including inflammatory reactions, cell proliferation, differentiation, cell adhesion and apoptosis (Karin et al., 2002). Thus, the regulatory role of RMRP on NF-κB p65 subunit potentiates this lncRNA as a contributor in many physiological and pathological processes.

An et al. (2020) have reported up-regulation of RMRP and Gadd45g in coronary atherosclerosis and human vascular smooth muscle cells, whereas miR-128-1-5P expression was decreased in these cells. RMRP silencing suppressed IL-6 and IL-8 production, and attenuated expression of apoptosis related proteins in these cells following ox-LDL treatment (An et al., 2020). Previous studies have indicated up-regulation of GADD45G in response to stressful growth arrest situations and exposure to DNA-damaging substances. In fact, GADD45G has an important role in response to environmental stress through facilitating activation of the p38/JNK pathway (Takekawa and Saito, 1998). Moreover, GADD45G has been shown to be regulated by NF-κB (Tamura et al., 2012). Thus, the interaction between RMRP, GADD45G and NF-κB might be involved in a wide variety of human disorders.

Expression of RMRP has also been increased in the model of ischemic cerebral injury. Notably, valproate has an inhibitory effect on RMRP expression, while increasing PI3K/Akt activity leading to enhancement of cell viability and attenuation of apoptosis (Li and Sui, 2020). Up-regulation of RMRP has also been observed in a number of immune-related conditions. For instance, RMRP expression has been increased in T cells of patients with rheumatoid arthritis in correlation with disease duration (Moharamoghli et al., 2019). Finally, expression of RMRP has been significantly down-regulated in patients with major depressive disorder compared with normal individuals in correlation with severity of depression. Besides, RMRP levels were decreased in an animal model of depression (Seki et al., 2019). Table 5 reviews the role of RMRP in non-cancerous pathologic conditions.

**TABLE 5:** Summary of RMRP studies in non-cancerous pathologic conditions.

**Pathologic conditions**	**Clinical samples**	**Cell lines**	**Animal models**	**Targets/regulators and signaling pathways**	**Results**	**References**
Sepsis (myocardial dysfunction)	–	Murine HL-1 cardiomyocytes	Male C57B6/L mice	miR-1-5p/HSPA4	RMRP regulated cardiomyocyte apoptosis and inhibited Lipopolysaccharide-induced sepsis.	Han et al. (2020)
Atherosclerosis	–	Human vascular smooth muscle cells, HEK293T	Wistar rats	miR-128-1-5P/Gadd45g	RMRP was upregulated in coronary atherosclerosis. Its downregulation associated with inhibition of IL-6, IL-8, and apoptosis related proteins.	An et al. (2020)
Ischemic heart failure (HF)	PBMC and left ventricle biopsies of 18 non-end-stage dilated ischemic cardiomyopathy and 17 controls/11 post-ischemic end-stage HF patients	–	C57BL/6 J male mice	–	RMRP was dysregulated in both end- and non-end-stage HF patients and mouse model of cardiac hypertrophy.	Greco et al. (2016)
Ischemic myocardial injury	–	H9c2	Sprague-Dawley male rats	miR-206/ATG3 and PI3K/AKT/mTOR pathway	RMRP downregulation enhanced cardiac function and suppressed apoptosis followed by myocardial I/R injury.	Kong et al. (2019)
Cardiac fibrosis	–	–	pathogen-free Sprague-Dawley rats	miR-613	RMRP was upregulated in cardiac fibrosis. Its reduction led to inhibition of cardiac proliferation, differentiation and collagen accumulation.	Zhang et al. (2019)
Ischemic stroke (IS)	–	BV-2	Adult male C57BL/6 mice	PI3K/Akt signaling pathway	Valproate inhibited RMRP expression, which increased survival rates and modulated cell apoptosis.	Li and Sui (2020)
Multiple sclerosis (MS)	Whole venous blood from 72 patients and 28 healthy controls	–	–	Th17 effector program	RMRP gene expression tended to be increased in patients with relapsing-remitting form of MS.	Ghaiad et al. (2020)
Rheumatoid arthritis (RA)	Peripheral blood from 20 RA patients and 18 healthy matched controls	–	–	–	RMRP expression was upregulated in T cells of RA patients and correlated with disease duration.	Moharamoghli et al. (2019)
Major depressive disorder (MDD)	Peripheral blood from 29 MDD patients and 29 matched healthy controls	–	Adult male BALB/c mice	–	RMRP was downregulated in MDD patients and mouse model. It was correlated with depression severity.	Seki et al. (2019)

## Discussion

Over-expression of RMRP has been reported in numerous types of cancers such as those originated from bladder, colorectal, lung, breast and gastric tissues. Moreover, up-regulation of RMRP is a marker of poor prognosis in these types of cancers. The results of *in vitro* and *in vivo* studies consistently verify this function for RMRP with a single exception in the hepatocellular carcinoma. In spite of vast mechanistical studies about the role of RMRP, the net situation of RMRP as a diagnostic marker in cancer has not been evaluated yet.

RMRP serves as a sponge for miR-206, miR-613, miR-1-3p, and miR-217. Among these miRNAs, the functional interaction between RMRP and miR-206 has been approved in different tissues. In addition to these miRNAs, expressions of tens of miRNAs have been altered following RMRP silencing, implying the vast extent of RMRP/miRNA network (Tang et al., 2019). Functional annotation of dysregulated miRNAs depicted their relevance with structural molecular functions, extracellular matrix elements, retinoid/isoprenoid binding, functions of cytokines and IFN-α/-β receptor binding (Tang et al., 2019). Almost all of these functions are related with the carcinogenesis process. Therefore, a possible route for participation of RMRP in this process is its interactions with miRNAs. One of the pathways being influenced RMRP is stem cells metabolism (Tang et al., 2019) which accords with the formerly reported role of RMRP in giving permission to cancer cells for infinite proliferation via interplay with TERT (Maida et al., 2009).

In addition to the mentioned malignant conditions, dysregulation of RMRP has been reported in ischemic myocardial injury, cardiac fibrosis, ischemic stroke, multiple sclerosis, rheumatoid arthritis and major depressive disorder. Evidence for contribution of RMRP in the pathogenesis of major depressive disorder has come from both human and rodent studies (Seki et al., 2019). Moreover decreased levels of this lncRNA in the circulation of patients potentiate it as a biomarker for this neuropsychiatric condition (Seki et al., 2019). The role of RMRP in construction of the nuclear RNase MRP complex and its function in the processing of ribosomal RNA, its effects in the regulation of epigenetic mechanisms and its interactions with the telomerase reverse transcriptase catalytic subunit have been suggested as possible mechanisms of participation of RMRP in major depressive disorder (Seki et al., 2019). Therefore, RMRP is involved in the development of diverse disorders. This finding is in accordance with the ubiquitous expression of RMRP in human tissues. Functionally, the majority of effects of RMRP in the pathogenesis of these disorders are explained by the regulatory role of this lncRNA in the mitochondrial functions particularly apoptotic pathways regulated by this organelle. In addition, based on the interaction between RMRT and miRNAs regulating cytokine activity, modulation of immune function is another mechanism of participation of RMRT in the development of these disorders especially rheumatoid arthritis and multiple sclerosis.

A major limitation of most of studies that assessed expression of RMRP in cancer patients is the small sample size and lack of validation of the obtained results in independent cohorts. Moreover, they have not assessed expression of this lncRNA in the circulation to unravel its potential as non-invasive biomarker.

Taken together, RMRP is an lncRNA whose dysregulation and somatic mutations have been demonstrated in solid and hematological malignancies. However, the association between the observed mutations and altered expression of this lncRNA has not been assessed yet. Thus, this field should be explored in future studies.

## Future Directions

Recent advances in high throughput sequencing techniques have facilitated identification of molecular counterparts of lncRNAs. Further attempts in this field would identify additional mRNA and miRNA molecules that function in upstream and downstream of RMRP, thus extending the functional network through which this lncRNA exerts its effects. Comprehensive assessment of these network would help in recognition of the most appropriate therapeutic targets for treatment of RMRP-associated disorders.

## Author Contributions

MT and SG-F wrote the draft and revised it. BH, TA, and HH collected the data and designed the tables and figures. All authors approved submitted version.

## Conflict of Interest

The authors declare that the research was conducted in the absence of any commercial or financial relationships that could be construed as a potential conflict of interest.
